# New insights for drug resistance in metastatic castration-resistant prostate cancer

**DOI:** 10.20517/cdr.2022.83

**Published:** 2022-08-02

**Authors:** Prem Prakash Kushwaha, Sanjay Gupta

**Affiliations:** ^1^Department of Urology, Case Western Reserve University, Cleveland, OH 44106, USA.; ^2^The Urology Institute, University Hospitals Cleveland Medical Center, Cleveland, OH 44106, USA.; ^3^Department of Pathology, Case Western Reserve University, Cleveland, OH 44106, USA.; ^4^Department of Pharmacology, Case Western Reserve University, Cleveland, OH 44106, USA.; ^5^Department of Nutrition, Case Western Reserve University, Cleveland, OH 44106, USA.; ^6^Divison of General Medical Sciences, Case Comprehensive Cancer Center, Cleveland, OH 44106, USA.

**Keywords:** Castration-resistant prostate cancer, drug resistance, cancer stem cells, androgen receptor, neuroendocrine differentiation, extracellular vesicles, chemotherapy, androgen receptor

## Abstract

Prostate cancer is the most common cancer and is the second leading cause of cancer-related deaths among men in the United States. Androgen deprivation therapy (ADT) is the standard treatment for advanced-stage prostate cancer; however, this treatment eventually fails, leading to an incurable disease subtype known as metastatic castration-resistant prostate cancer (mCRPC). There are several molecular mechanisms that facilitate the development of mCRPC engaging androgen receptor (AR) growth axis, including AR amplification, gain of function AR mutations, and AR splice variants that are constitutively active and are a foremost factor for mCRPC development. AR-independent mechanisms with exceptionally low or absent AR expression found in cancer cells suppress ADT effectiveness and contribute to aggressive variants, including neuroendocrine differentiation. Several other AR regulatory factors such as epigenetic modification(s), and DNA damage response have been reported during post-ADT exposure and play a crucial role in mCRPC development. Therefore, targeting prostate cancer cells before their progression to mCRPC would improve patient outcomes. This special issue in “Cancer Drug Resistance” focuses on understanding the mechanism(s) and development of mCRPC resistance. This special issue also highlights the therapeutic strategies to combat against resistant subtype. This issue comprehensively reviews the mCRPC and delivers the update in the forum of mCRPC resistance development.

## INTRODUCTION

In recent years, studies related to metastatic castration-resistant prostate cancer (mCRPC) have received global attention. mCRPC is an aggressive and advanced form of prostate cancer, having progressed to various organ sites and no longer responding to androgen deprivation therapies that result in lowering of testosterone^[1-2]^. Although several treatment options are available for mCRPC patients, the disease remains incurable, with a poor prognosis and low survival rates^[3]^. Current systemic treatment options for mCRPC include hormonal therapy, chemotherapy, immunotherapy, radiotherapy, and bone-modifying agents^[4]^. There are few recently included novel therapies such as PARP inhibitors and immune checkpoint inhibitors including PD-1/PD-L1 and CTLA4 for a subset of mCRPC patients^[5]^. The special issue of “Drug resistance in metastatic castration-resistant prostate cancer” provides insights into the targets and treatment of mCRPC.

## ARTICLE DESCRIPTION

In the present special issue, a total of six articles have been qualified and published. The first review published by Yehya *et al*. (2022) shed the light on emerging targeted therapies currently evaluated in clinical trials with promising potential to overcome mCRPC-drug resistance^[6]^. This review also provides the updated mechanism of action of mCRPC development. The authors highlight the role of cytochrome P450 enzyme 17 (CYP17) in steroidal biosynthesis, especially androgen synthesis. Implications of altered cellular signaling pathways, kinases, and other factors such as cytokine and enzymes that endorse the mCRPC resistance are described in this review. The authors also highlight the completed and ongoing clinical trials against mCRPC, including PARP inhibitors, immunotherapies, AR degraders, and PI3K signaling inhibitors.

Another original article by Kumar *et al*. (2022) showed the role of extracellular vesicles secretion in paclitaxel resistance of prostate cancer cells^[7]^. Treatment of paclitaxel (PTX) resistant prostate cancer cells with GW4869 significantly reduced the release of small EVs (50-100 nm size range) while increasing the release of larger EVs (> 150 nm in size) and inhibited their clonogenicity. Moreover, GW4869 treatment significantly inhibited the survival of PTX-resistant prostate cancer cells in a dose-dependent manner and reduced the tumor weight in an *in vivo *model.

Kushwaha *et al*. (2022) performed a critical review and demonstrated the role of prostate cancer stem-like cells (PCSCs) in the development of antiandrogen resistance^[8]^. PCSCs have the ability to differentiate, self-renew, and regenerate tumor heterogeneity. A variety of factors contribute to increasing PCSCs stemness including AR variants, AR mutation, and epigenetic and genetic alterations that lead to changes in the tumor microenvironment, such as adenosine 5′-triphosphate (ATP)-binding cassette (ABC) transporters expression and molecular signaling pathways. This review explored the molecular pathways in the generation of prostate cancer stem-like cells post ADT, various PCSCs markers, and a list of genes, their type and location along with fold change values associated with hematopoiesis pluripotent stem cells. The methods for isolation, identification, and characterization of PCSCs from primary prostate tumors have also been described.

Choi *et al*. (2022) provided a perspective on the transcription factor ONECUT2 which mediates AR-independent cell growth and neuroendocrine differentiation in CRPC^[9]^. It is a key factor in NE differentiation between adenocarcinoma to neuroendocrine prostate cancer (NEPC). The perspective specified genes including AR regulatory genes, FOXA1/2, and hepatocyte nuclear factor (Hnf) are the target(s) of ONECUT1 and ONECUT2. Specifically, ONECUT2 regulates the signaling of hypoxia-inducing factors and consequently promotes prostate cancer differentiation and can also serve as a therapeutic target in mCRPC.

Khalil *et al*. (2022) summarize the multifaceted role of Tousled-like kinase 1 (TLK1) in mCRPC progression and development of therapeutic resistance^[10]^. The highlight of this review includes that DDR kinase TLK1 mediates an important aspect of adaptation to androgen deprivation and promotes cell cycle progression under unfavorable growth conditions such as ADT and reprograms prostate cancer cells to adapt to androgen-independent growth and resistance development.

The final review article by Biersack *et al. *(2022) demonstrated that epigenetic modification of histone deacetylase (HDAC) plays a significant role in different mechanisms contributing to the development and persistence of CRPC chemoresistance^[11]^. The clinical trials on HDAC inhibitors for the treatment of CRPC were reviewed and the major outcomes have been summarized. Several other new HDAC inhibitors and the effect of HDAC inhibitors in combination therapies using anticancer drugs were highlighted.

Altogether, this special issue is a comprehensive review of the mechanism(s) of drug resistance and additional therapeutic opportunities for mCRPC treatment.

## DECLARATIONS

### Copyright

@ The Author(s) 2022.
